# Assessment of the Impact of Post-Thaw Stress Pathway Modulation on Cell Recovery following Cryopreservation in a Hematopoietic Progenitor Cell Model

**DOI:** 10.3390/cells11020278

**Published:** 2022-01-14

**Authors:** John M. Baust, Kristi K. Snyder, Robert G. Van Buskirk, John G. Baust

**Affiliations:** 1 CPSI Biotech, 2 Court St., Owego, NY 13827, USA; ksnyder@cpsibiotech.com (K.K.S.); rvanbus@binghamton.edu (R.G.V.B.); 2 Center for Translational Stem Cell and Tissue Engineering, Binghamton University, 4400 Vestal Parkway East, Binghamton, NY 13902, USA; jgbaust@binghamton.edu; 3 Department of Biological Sciences, Binghamton University, 4400 Vestal Parkway East, Binghamton, NY 13902, USA

**Keywords:** cryopreservation, apoptosis, delayed onset cell death, recovery, viability, *RevitalICE*

## Abstract

The development and use of complex cell-based products in clinical and discovery science continues to grow at an unprecedented pace. To this end, cryopreservation plays a critical role, serving as an enabling process, providing on-demand access to biological material, facilitating large scale production, storage, and distribution of living materials. Despite serving a critical role and substantial improvements over the last several decades, cryopreservation often remains a bottleneck impacting numerous areas including cell therapy, tissue engineering, and tissue banking. Studies have illustrated the impact and benefit of controlling cryopreservation-induced delayed-onset cell death (CIDOCD) through various “front end” strategies, such as specialized media, new cryoprotective agents, and molecular control during cryopreservation. While proving highly successful, a substantial level of cell death and loss of cell function remains associated with cryopreservation. Recently, we focused on developing technologies (*RevitalICE*™) designed to reduce the impact of CIDOCD through buffering the cell stress response during the post-thaw recovery phase in an effort to improve the recovery of previously cryopreserved samples. In this study, we investigated the impact of modulating apoptotic caspase activation, oxidative stress, unfolded protein response, and free radical damage in the initial 24 h post-thaw on overall cell survival. Human hematopoietic progenitor cells in vitro cryopreserved in both traditional extracellular-type and intracellular-type cryopreservation freeze media were utilized as a model cell system to assess impact on survival. Our findings demonstrated that through the modulation of several of these pathways, improvements in cell recovery were obtained, regardless of the freeze media and dimethyl sulfoxide concentration utilized. Specifically, through the use of oxidative stress inhibitors, an average increase of 20% in overall viability was observed. Furthermore, the results demonstrated that by using the post-thaw recovery reagent on samples cryopreserved in intracellular-type media (Unisol™), improvements in overall cell survival approaching 80% of non-frozen controls were attained. While improvements in overall survival were obtained, an assessment on the impact of specific cell subpopulations and functionality remains to be completed. While work remains, these results represent an important step forward in the development of improved cryopreservation processes for use in discovery science, and commercial and clinical settings.

## 1. Introduction

At no time in history has cryopreservation played as critical a role as an enabling technology than today. From basic research to pharma discovery science, cell therapy, regenerative medicine, tissue engineering, vaccines, endangered species conservation, and organ transplantation, cryopreservation serves as an enabling technology, allowing for a transient halting of biological time [1,2]. This, in turn, facilitates broad-based production, storage, and transport of living systems. For instance, according to clinicaltrials.gov, cryopreserved products are now used in over 1000 clinical trials involving cell therapy, CAR-T cells, vaccines, stem cells, and engineered tissues worldwide. In many, if not all, of these cases, the successful utilization of said therapy hinges upon cryopreservation. 

While cryopreservation is typically viewed as a tool (step) in a larger process, cryopreservation itself is in fact a rate-limiting step. Low post-thaw yields (recovery), compromised biologic function, and inability for systems to regenerate resulted in many groups shying away from cryopreservation when possible, relegating cryopreservation primarily to the academic and research areas [1,2]. The result was that the development of new life saving biologic products was stifled, as they could only be stored for a few hours to days. This significantly limited product distribution, limiting these biologic products to “just in time” use status. This remained the status quo from the late 1950s, when the cryoprotective properties of dimethyl sulfoxide (DMSO) was reported by Lovelock and Bishop [3], through the 1990s, despite decades of research, millions of dollars spent, and numerous groundbreaking discoveries in the cryobiological sciences by Mazur, Meryman, Steponkus, Fahy, Peg, Toner, Liebo, and McGann, among others [4,5,6,7,8,9,10,11,12,13,14,15,16,17,18,19,20]. This changed in 1998 when Baust et al. first reported on the involvement of a complex molecular biological stress response of cells to the freeze−thaw process [21,22]. This molecular response was found to manifest in the activation of apoptotic and secondary necrotic processes, ultimately leading to cell death hours to days after thawing, a phenomena termed cryopreservation induced delayed onset cell death (CIDOCD) [22,23,24,25]. Studies by this group, as well as others, also demonstrated that by controlling this stress response, one could improve not only cell survival, but also cell function and repopulation post-thaw [22,23,25,26,27,28,29,30,31,32,33,34,35,36]. Furthermore, it was found that this molecular control strategy could reduce the need for traditional cryoprotective agents (CPA) such as DMSO [37,38]. This discovery resulted in a paradigm shift in the cryopreservation sciences from a primarily chemo-osmometric (ice control) approach to that of an integrated approach combining molecular biological control with ice control [24,39,40,41,42]. 

While many researchers remain unaware, today, this shift is mainstream and serves as the foundational basis of today’s commercial intracellular-like cryopreservation media such as CryoStor^®^ and Unisol™ [40,41,42,43,44,45,46,47,48,49]. Functionally, through their multi component formulation, intracellular-like solutions buffer the environment a cell is exposed to during the freeze−thaw process, modulating stress response activation, thereby reducing CIDOCD [23,25,41,42,50]. Numerous reports have detailed the benefits of using intracellular-like solutions in comparison to traditional extracellular-like cell culture media with DMSO [22,46,47,51,52,53,54,55,56]. Today, 100’s of studies have demonstrated >50% improvement in cell recovery and function through the use of intracellular-like solutions in an equal number of unique cell and tissue systems, ranging from stem cells to CAR-T cells to engineered tissues. While taking over a decade for this shift to become an integral part of the cryopreservation sciences, intracellular-like solutions are now the gold standard for modern cryopreservation [41,42,44,45,54,55,57]. 

While the integration of cellular−molecular control and the introduction of intracellular-like cryopreservation solutions has had a transformative impact providing for significant increases in the recovery, repopulation, function, and structural integrity of cells and tissues (native and engineered) post-thaw, several challenges remain to enable an even greater impact. These include (1) continued cell loss (ranging from 10 to >50% depending on the cell type), (2) compromised function in complex cells and tissues, (3) spontaneous differentiation in native stem cell populations, (4) dependence on DMSO, and (5) lack of/limited utility of equipment used in the freezing and thawing processes [1,2,41,54,55,56,57,58]. Furthermore, recognition of the critical role cryopreservation plays in the success of the field of regenerative medicine remains a hurdle. In an effort to address these challenges and improve the outcome, today’s researchers are focused on developing new CPAs [59,60,61,62,63,64,65,66,67,68], targeted apoptotic control [24,25,26,27,28,29,30,31,32,33,34] during cryopreservation and new devices [69,70,71]. On the discovery science side, groups continue to investigate the pathways activated during cryopreservation, as well as during the recovery phase [72,73,74,75,76,77]. In this regard, our group has recently focused on investigating the molecular stress response pathways activated following thawing, and if this stress response can be modulated post-thaw to improve cell survival. Our hypothesis is that controlling the extent of stress response activation post-thaw will result in an increase in cell survival. Furthermore, we hypothesize this benefit can be obtained regardless of the cryopreservation conditions utilized (freeze media, CPA, freeze/thaw protocol, etc.). If successful, this would impact not only the ever-advancing field of regenerative medicine, which now relies on intracellular-like solutions, but could also impact the tens of millions of “banked” samples cryopreserved years ago using traditional approaches. This study was designed to focus on the issue of continued cell loss experienced following cryopreservation. This is an important first phase in improving outcomes, as once improved survival is obtained, then studies focused on second level issues (elimination of DMSO, cell function, etc.) would be justified. Accordingly, in this study, we investigated the impact of modulating several stress response pathways following cryopreservation in both traditional extracellular-like (culture media) and intracellular-like (Unisol™) cryopreservation freeze media with and without DMSO supplementation. Specifically, we investigated the impact of targeting apoptotic caspase activation, oxidative stress, unfolded protein response, and free radical damage in the initial 24 h post-thaw on overall cell survival (recovery) using a human hematopoietic progenitor cell (hHPC) model in vitro. 

## 2. Materials and Methods

### 2.1. Cell Culture

Human hematopoietic progenitor (hHPC) cells (Mobilized Peripheral Blood CD34+ Stem/Progenitor Cells (M34C-GCSF-1) HemaCare, Los Angles, CA, USA) were maintained under standard culture conditions (37 °C, 5% CO_2_/95% air) in an hHSC basal medium supplemented with a low serum growth kit, gentamicin, amphotericin B, penicillin, and streptomycin (StemSpan SFEM II media with StemSpan™ CD34+ Expansion Supplement, StemCell Technologies, Vancouver, BC, USA). Cells were propagated in T-75 flasks (CellTreat, Shirley, MA, USA) and the media were replenished every two days of cell culture. 

### 2.2. Cryopreservation

The cell culture medium was replaced with fresh growth medium 24 h prior to freezing. The loosely adhered cell culture was detached by the addition of TrypLE™ (Invitrogen, Waltham, MA, USA) for 2 min at room temperature. After incubation, 5 mL of growth medium was added to the cell suspension to inhibit enzymatic dissociation. The cell suspension was divided equally and was pelleted at 100× *g* for 5 min. The supernatant was decanted, and the cell pellet was resuspended at 1 × 10^6^ cells/mL in the various cryopreservation solutions at 10 °C. The cryopreservation solutions utilized consisted of culture media (StemCells) or Unisol™ (Tissue Testing Technologies LLC., North Charleston, SC, USA) supplemented with 0, 5 or 10% DMSO (Sigma, St. Louis, MO, USA). The Unisol™ solution was supplemented with 3 mM Glutathione (Glu) prior to use, as per the manufacturer’s recommendation. The cell suspensions were then placed into cryovials (1 mL/vial) and were frozen under a standard controlled rate protocol of ~−1 °C min^−1^ to −80 °C and stored at liquid nitrogen (LN_2_) temperatures (−196 °C). Following storage, cells were rapidly thawed using SmartThaw™ (CPSI Biotech, Owego, NY, USA) until the ice dissipated and samples were cold (~0 °C). A one-step dilution (1:12) in a complete growth medium was performed, then 100 μL of cell suspension was plated onto individual wells of a 96-well tissue culture plate (TrueLine, MedSupply Partners, Atlanta, GA, USA) and placed into standard culture conditions (37 °C, 5% CO_2_) for recovery and assessment. The growth medium was replenished in the cultures at 24-h intervals for the cell survival studies. 

### 2.3. Modulation Studies

Chemical modulation of the molecular pathways was conducted through the use of salubrinal (UPR-specific inhibitor), resveratrol (free radical scavenger), n-acetyl cystine (oxidative stress inhibitor), and pan caspase inhibitor (apoptotic inhibitor). Salubrinal (EMD Chemicals Inc., Gibbstown, NJ, USA) was added to the recovery media (*RevitalICE*™, CPSI Biotech) at a working concentration of 25 µM, resveratrol (EMD Chemicals Inc.) at 11 µM, n-acetyl cystine (Sigma), and caspase inhibitor (Calbiochem) at 10 µM, immediately before utilization. These concentrations were selected based on previous studies in other cell systems, in addition to dose response experiments (data not shown). Chemicals were diluted in DMSO prior to utilization with final working concentrations of DMSO at 1.3 µM (0.01%) for resveratrol and caspase inhibitor, and 32 µM (0.25%) for salubrinal. N-acetyl cysteine was diluted in culture media. DMSO controls of 1.3 µM and 32 µM were conducted to assure no effect of the dilution vehicle. 

### 2.4. Cell Viability

#### 2.4.1. Metabolic Activity 

To assess cell viability, the metabolic activity assay alamarBlue™ (Invitrogen) was utilized. The cell culture medium was aspirated from the 96-well plates and 100 µL/well of the working alamarBlue™ solution (1:20 dilution in HBSS with Ca++ and Mg++ without phenol red, Corning/MediaTech, Manassas, VA, USA) was applied. Samples were then incubated for 60 min (±1 min) at 37 °C in the dark. The fluorescence levels were analyzed using a Tecan SPECTRAFluorPlus plate reader (TECAN, Austria GmbH, Vienna, Austria). Relative fluorescence units were converted to a percentage compared to normothermic controls set at 100% and were data graphed using Microsoft Excel. Viability measurements were taken at 1 and 24 h of recovery. 

#### 2.4.2. Fluorescence Microscopy

Assessment of the cell membrane integrity was also conducted 24 h post-thaw using calcein-AM (Invitrogen) on replicate samples. The cell culture medium was aspirated from the 96-well plates and 100 µL/well of the working solution of Calcein-AM (1:500 dilution in HBSS with Ca++ and Mg++ without phenol red) was added to each well. Samples were incubated in the dark for 30 min and then fluorescence images were obtained using a Zeiss Axiovert 200 fluorescent microscope with the AxioVision 4 software (Zeiss, Jena, Germany). 

### 2.5. Data Analysis 

Viability experiments were repeated a minimum of three times with an intra-experiment repeat of seven replicates, yielding a total replicate of *n* > 21 (e.g., N > 3; *n* > 21). Fluorescence microscopy was conducted on a minimum of three replicate wells from a minimum of three separate experiments. Data from all individual experimental replicates (*n* > 21) were combined, averaged, and compared to the normothermic controls. Means and standard deviations (SD) were calculated using the combined data set (inter and intra experimental values), and single-factor analysis of variance (ANOVA) and Student’s *t*-tests were utilized to determine statistical significance. Statistical significance (*p*-values) is reported within text. 

## 3. Results

### 3.1. Assessment of the Post-Thaw Assessment Interval

**Figure 1:**
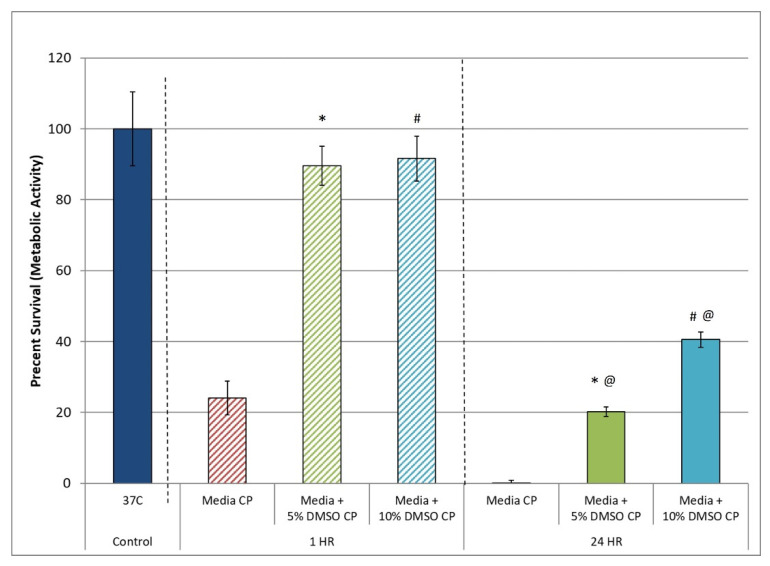
Comparison of hHPC cell survival levels (Metabolic Activity) at 1 h vs. 24 h post-Thaw. Human hematopoietic progenitor cells were cryopreserved in media with 5% or 10% DMSO, and assessed for viability at 1 and 24 h post-thaw. Data show a substantial level of cell loss over the initial 24 h recovery period and that the 24 h assessment point gave a clearer indication on overall cell survival. Means (±SD) were calculated by combining the individual replicates for all experiments (*n* > 21). (*, # and @ = *p* < 0.01).

Assessment of cell survival following cryopreservation immediately post-thaw (within 1 h) with dye exclusion assays (trypan blue or similar) remains mainstream today. This is despite the fact that numerous reports have demonstrated a tremendous loss of cells within the initial 24 h post-thaw period [21,37,38]. As described elsewhere, this cell loss (termed CIDOCD) has been shown to be a result of the delayed activation of apoptotic and necrotic cascades during the initial recovery interval [23,24,25]. These studies have illustrated the importance of assessing survival with multiple assays and/or at multiple time points post-thaw to gain a true understanding of survival. As such, in this study, we assessed hHPC survival at 1 and 24 h following thawing to determine the extent of CIDOCD associated with hHPC cryopreservation. Analysis of hHPC samples at 1 h post-thaw revealed a high level (>90%) of viability (metabolic activity) in samples cryopreserved in culture media + 5% and 10% DMSO (M5 and M10, respectively; Figure 1). Assessment of replicate samples at 24 h post-thaw revealed a marked decline in hHPC viability to 26.4% and 38.4% in the M5 and M10 samples, respectively. Specifically, hHPC survival was found to decrease from 92.2% (±5.6) to 20.8% (±1.3) (*p* < 0.01(*)) in M5 samples, and from 94.3% (±6.4) to 41.7% (±2.2) in M10 samples (*p* < 0.01(#)). When comparing the overall survival of the M5 and M10 samples, it was found that the 10% DMSO solution (M10) yielded a significant increase in overall survival compared to M5 (41.7% vs. 20.8%, respectively, *p* < 0.01(@)). The observed decline in survival over the initial 24 h period was consistent with previous reports; therefore, the 24 h post-thaw time point was utilized as the primary assessment point. 

### 3.2. Comparison of Extracellular vs. Intracellular Freeze Media

**Figure 2:**
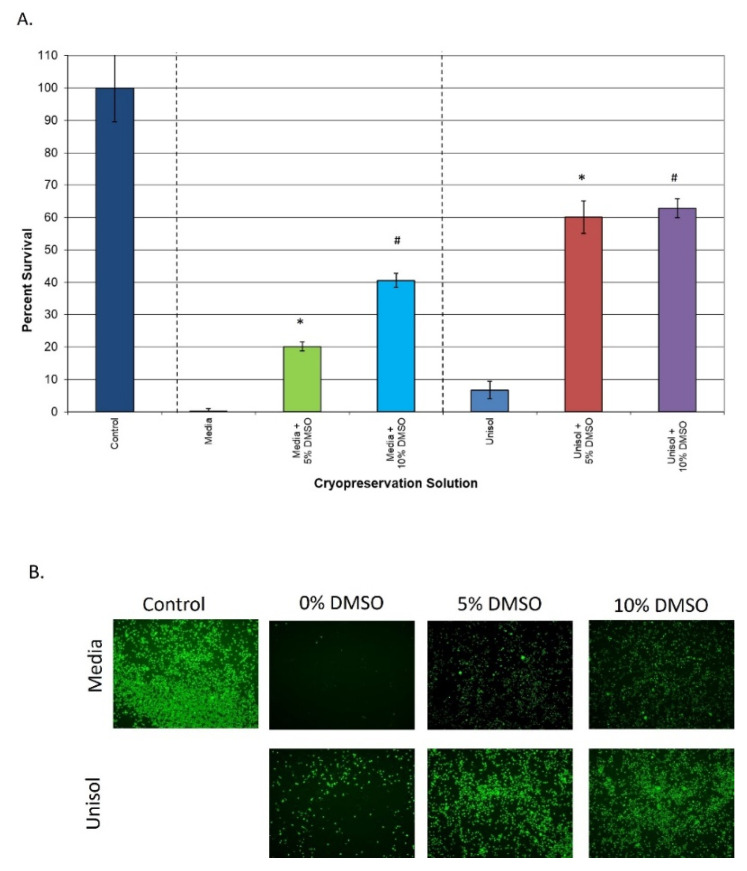
Comparison of hHPC cell 24 hour post-thaw survival following cryopreservation in various cryopreservation solutions. Human hematopoietic progenitor cells were cryopreserved in extracellular-type (media) or intracellular-type (Unisol™) cryopreservation media with 0%, 5%, or 10% DMSO and assessed for viability at 24 h post-thaw. (**A**) Post-thaw metabolic activity assessment revealed that cryopreservation in Unisol™ + DMSO yielded a significant increase in overall viability (* and # = *p* < 0.01). Means (±SD) were calculated by combining the individual replicates all experiments (*n* > 21). (**B**) Post-thaw fluorescent imaging using Calcein-AM confirmed the improvement in cell survival with an increased DMSO concentration, as well as in the Unisol™ + CPA samples.

With the establishment of the 24 h post-thaw time point and the culture media-based cryopreservation solutions providing <42% overall survival, studies were conducted to evaluate the effect of the intracellular-type cryopreservation solution Unisol. To this end, hHPC samples were cryopreserved in Unisol™ + 5% DMSO (U5) and 10% DMSO (U10), and the 24 h post-thaw viability was compared to that of M5 and M10 (Figure 2). Analysis of hHPC samples cryopreserved with U5 and U10 revealed a post-thaw viability of 60.1% (±5.7) and 62.9% (±2.9), respectively (Figure 2A). In comparison to the media condition, the U5 samples demonstrated a three-fold increase in overall viability vs. M5 (60.1% vs. 20.8%, *p* < 0.01(*)), whereas the U10 samples yielded a 1.5-fold increase over M10 samples (62.9% vs. 41.7%, *p* < 0.01(#)). Interestingly, despite the significant improvement in overall viability in Unisol™ with DMSO, for the samples compared to matched media + DMSO samples, only a ~2% difference in overall viability was observed between the U5 and U10 samples (*p* = 0.64). This differed from the M5 and M10 samples, where the increase in DMSO concentration from 5% to 10% resulted in a two-fold increase in post-thaw viability (20% vs. 41%, respectively). Replicate samples were also assessed with the membrane integrity dye Calcein-AM via fluorescence microscopy to confirm or refute the metabolic activity data. Analysis of the fluorescence images revealed an increase in cell number between the M5 and M10 samples, and a further increase in the U5 and U10 samples (Figure 2B). These findings correlated well with the metabolic activity viability data. 

### 3.3. Assessment of the Impact of Post-Thaw Cell Stress Modulation

With the establishment of a significant level of hHPC cell loss following cryopreservation (>60% with media + DMSO and ~40% Unisol™ + DMSO), coupled with the identification of a significant portion of the cell loss manifesting over the initial 24 h post-thaw period, we investigated the impact of modulating cell stress response pathways during the initial recovery period. This was conducted in an effort to further increase hHPC post-thaw recovery. These studies were initiated as the observed delayed cell loss (CIDOCD) has been reported to be a result of the activation of stress response pathways following thawing, manifesting in delayed apoptosis and necrosis 24 to 48 h post thaw in numerous cell systems [22,23,24,25,26,27,28,29,30,31,32,33,34]. Studies by our group and others have reported the involvement of the apoptotic caspase cascade (A), unfolded protein response (UPR), mitochondrial oxidative stress response (OSR), and free radical damage (reactive oxygen species (ROS)) resulting in the manifestation of CIDOCD [22,23,24,25,35,50,78]. We investigated the impact of modulating these pathways with the chemical modulators salubrinal (UPR I), resveratrol (ROSI), n-acetyl cystine (OSRI), or caspase inhibitor (AI) during the initial 24 h recovery period in samples cryopreserved in both media- and Unisol™-based solutions. 

#### 3.3.1. Impact on Samples Cryopreserved in Extracellular Freeze Media

**Figure 3:**
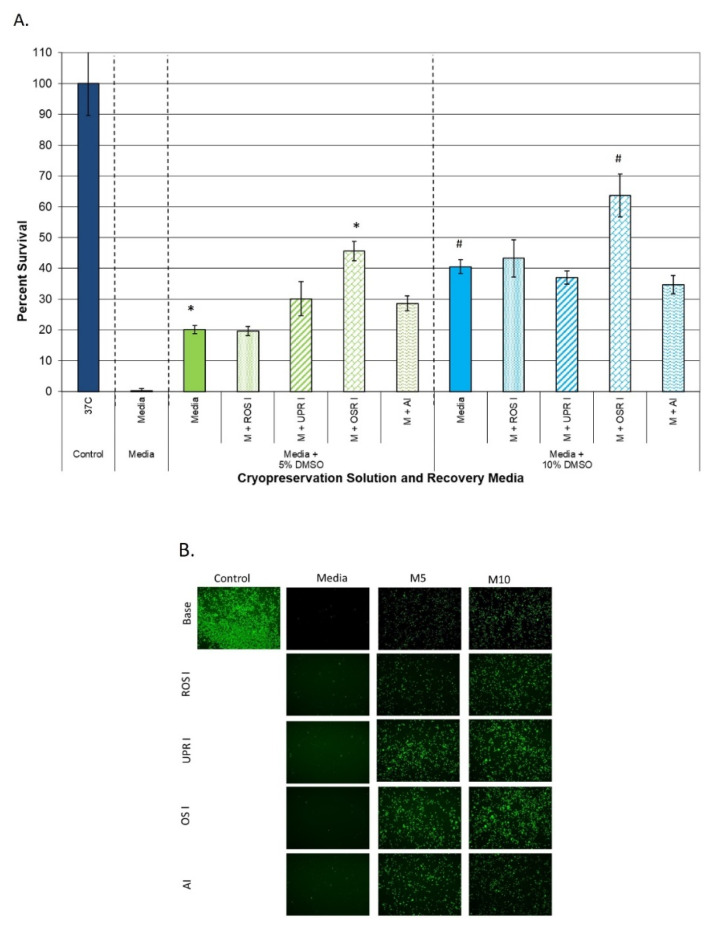
Human hematopoietic progenitor cells cryopreserved using a extracellular-type cryopreservation media (culture media) were incubated in recovery media supplemented with various cell stress modulators and assessed for viability at 24 h post-thaw. (**A**) Post-thaw metabolic activity assessment revealed that post-thaw incubation with the oxidative stress response inhibitor (OSR I) yielded an increase in overall viability (* and # = *p* < 0.01). Means (±SD) were calculated by combining the individual replicates of all of the experiments (*n* > 21). (**B**) Post-thaw fluorescence imaging using Calcein-AM confirmed the improvement in cell survival following oxidative stress response inhibitor conditioning for the initial 24 h post-thaw recovery interval.

When examining the media + DMSO conditions, post-thaw incubation with resveratrol (ROS inhibitor) for the initial 24 h recovery interval revealed no significant benefit in either of the media + DMSO conditions compared to the untreated M5 and M10 samples (Figure 3A, ROS I). When salubrinal (UPR inhibitor (UPR I)) or the caspase inhibitor (AI) was incorporated into the recovery media, a ~10% increase in 24 h post-thaw viability was observed in the M5 samples (30.1% (±5.6) and 29.1% (±2.3), respectively, vs. 20.2% for M5, *p* < 0.01) (Figure 3A, UPR I and AI). Interestingly, when applied to M10 samples, neither salubrinal nor caspase inhibitor supplementation to the recovery media had any benefit (37.0% (±2.1) and 35.1% (±2.9), respectively, vs. 40.6% (±2.2) for M10, *p* > 0.01). In contrast, when the recovery media were supplemented with n-acetyl cystine (oxidative stress response inhibitor (OSR I)), a significant increase in post-thaw viability was observed in both the M5 and M10 samples (Figure 3A, OSR I). Specifically, n-acetyl cystine supplementation yielded an overall survival of 45.6% (±3.1) in M5 samples and 63.7% (±6.9) in M10 samples. This represented an overall increase in viability of ~25% in M5 samples (20.2% vs. 45.6%, *p* < 0.01(*)) and a ~20% overall increase in M10 samples (43.2% vs. 63.7%, *p* < 0.01(#)). Post-thaw assessment with Calcein-AM via fluorescence microscopy visually confirmed the increase in viable cell number at 24 h post-thaw following incubation in recovery media supplement with n-acetyl cystine compared to the other conditions (Figure 3B).

#### 3.3.2. Impact on Samples Cryopreserved in Intracellular Freeze Media

**Figure 4:**
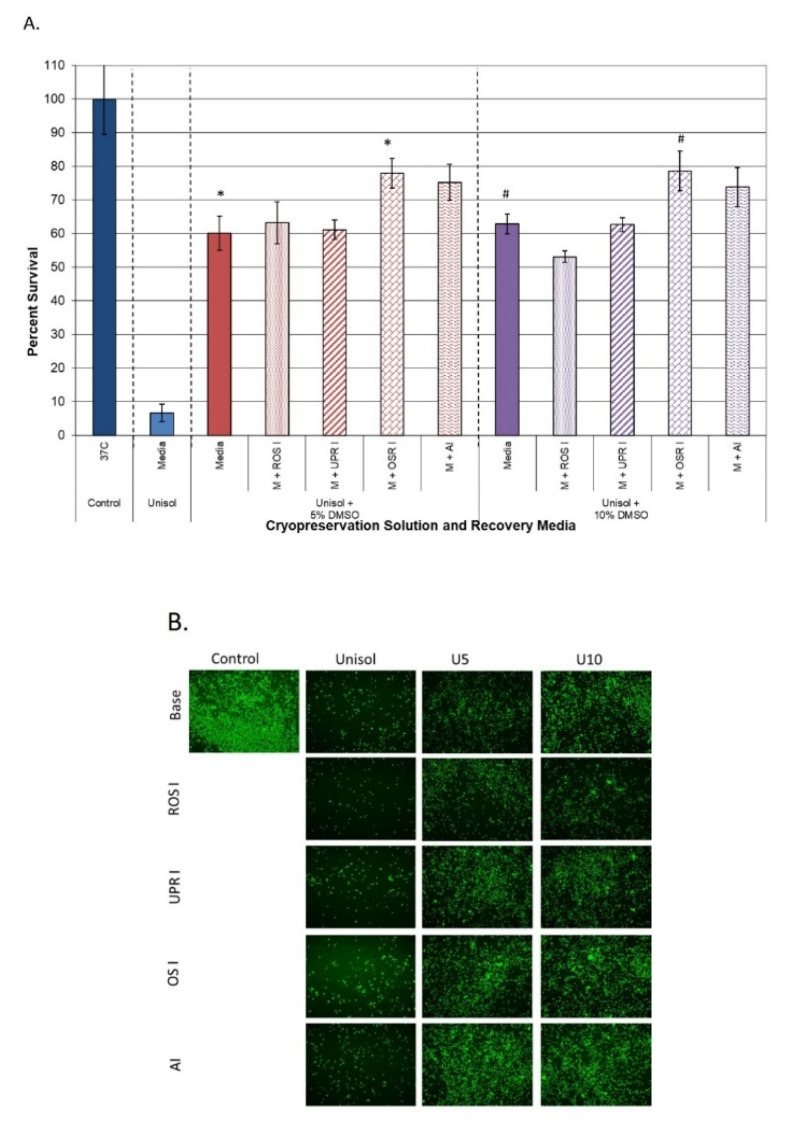
Assessment of the impact of post-thaw conditioning on hHPC cell viability following cryopreservation using intracellular-type media with DMSO. Human hematopoietic progenitor cells cryopreserved using a intracellular-type cryopreservation media (Unisol™) were incubated in the recovery media supplemented with various cell stress modulators and were assessed for viability at 24 h post-thaw. (**A**) Post-thaw metabolic activity assessment revealed that post-thaw incubation with the oxidative stress response inhibitor (OSR I) and apoptotic inhibitor (AI) yielded a significant increase in overall viability (* and # = *p* < 0.01). Means (±SD) were calculated by combining the individual replicates of all of the experiments (*n* > 21). Specifically, the combination of Unisol™ + DMSO and post-thaw conditioning with OSR I or AI resulted in an increase in overall viability > 78%. (**B**) Post-thaw fluorescence imaging using Calcein-AM confirmed the improvement in cell survival following oxidative stress response inhibitor or apoptotic inhibitor conditioning for the initial 24 h post-thaw recovery interval.

Recovery media supplementation in the Unisol™ + DMSO cryopreserved samples yielded a similar outcome to that of the media + DMSO samples. As with the M5 and M10 samples, recovery media supplementation with resveratrol conferred no benefit in either the U5 or U10 samples (Figure 4A, ROS I). Additionally, when salubrinal (UPR I) was incorporated into the recovery media, no benefit was noted in the Unisol™ samples (Figure 4A, UPR I). Unlike the media + DMSO samples, when the caspase inhibitor was added to the recovery media, a significant increase in viability was noted in both the U5 and U10 samples. Specifically, caspase inhibition resulted in a ~15% increase in overall viability in the U5 samples (75.3% (±5.3) vs. 60.1% (±5.1), respectively, *p* < 0.01) and a ~11% in U10 samples (73.8% (±5.8) vs. 62.9% (±2.9), respectively, *p* < 0.01). When n-acetyl cystine was added to the recovery media, a further increase in post-thaw viability was observed. In the U5 samples, N-acetyl cystine supplementation resulted in an ~18% increase, yielding an overall post-thaw viability of 78.0% (±4.5) vs. 60.1% (±5.1) for the non-supplemented U5 samples (*p* < 0.01(*)). A similar increase in overall viability to 78.8% (±5.9) was also observed in the n-acetyl cystine U10 samples. This represented a ~16% overall increase compared to non-supplemented U10 samples (*p* < 0.01(#)). As with the parent U5 and U10 conditions, no significant difference in post-thaw viability was observed between the U5 and U10 samples recovered in n-acetyl cystine (*p* = 0.87). Post-thaw assessment via fluorescence microscopy using Calcein-AM visually confirmed the increase in viable cell number in the U5 and U10 samples following a 24 h incubation period in recovery media supplemented with n-acetyl cystine and caspase inhibitor compared to the other recovery media conditions (Figure 4B). Initial observations of the number of CD34+ cells within the U5 and U10 samples cultured in recovery media with and without n-acetyl cystine suggest similar overall fractional percentages within each of the respective surviving populations (>80% of the viable population). While promising, these observations are preliminary and, as such, further investigation is necessary before any conclusions can be drawn.

## 4. Discussion

The development and use of complex cell-based products in clinical and discovery science continues to grow at an unprecedented pace. While playing a critical enabling role, cryopreservation also presents numerous challenges to the process [42,49,54,56,79]. To this end, there is no greater issue than the extensive cell death following cryopreservation, which results in compromised cell product quality. This negative impact on product quality can have significant downstream effects on cell function, delivery dosage, product costing, therapeutic efficacy, etc. [1,2,58,80,81,82,83,84,85,86,87,88]. For example, a given therapy requires a specific dose (cell number) to be effective. Once the dose is determined, vials are prepared and cryopreserved. If upon thawing there is a loss of 40–50% of the cells, the delivered dose would be half of that anticipated, and would ultimately be ineffective. Similar scenarios can have vast impacts on drug development, discovery science, tissue engineering, and other areas where cryopreserved cell products are utilized. Furthermore, if these cells that are undergoing both necrosis and apoptosis are delivered systemically in stem cell therapy applications, they could launch an unwanted immune response. Given this, it is paramount for individuals cryopreserving and utilizing cryopreserved cell products to recognize, account for, and control the cell loss associated with their specific cell system and cryopreservation protocol. It is now recognized that assessment of the sample viability at 24 h post-thaw using multiple viability assays is more appropriate [42,88,89,90]. Furthermore, once the overall survival is determined, incorporation of a functional assay into the assessment protocol at 24 h or longer (cell type specific) is encouraged [22,42,90,91]. In addition, tight control of the cryopreservation freezing and thawing process are also critical [4,11,18,40,42,69,71]. 

Over the last decade, numerous studies have investigated the impact of CIDOCD on various cell systems, as well as the benefit of controlling the activation of this molecular response through the use of specialized intracellular-type cryopreservation media [41,43,44,46,47,48,55,56,57,92,93]. Furthermore, studies have also demonstrated that the incorporation of molecular pathway modulators such as caspase, ROCK, calpain inhibitors, among others, into cryopreservation media can also increase overall viability in select systems [24,25,26,27,28,29,30,31,32,33,34]. While beneficial, in many cases, alteration or modification of the cryopreservation solution is not a plausible route to improve outcome. These include cases wherein production lots of cell products have already been cryopreserved (banked) and cases where GMP protocols have already been, or are in the process of being, validated, among others. In these scenarios, a change in the cryopreservation solution would require months to years of research, testing, and validation work, all of which would impact any previously produced inventories, potentially obsoleting millions of dollars of life saving products. As such, shifts in the front end of the cryopreservation process are often not feasible.

In this study, we investigated modulating the molecular stress response to cryopreservation during the post-thaw recovery period (back end of the cryopreservation process) as a means of increasing cell recovery 24 h post-thaw. Here, our studies focused on analyzing the impact of the inhibition of the unfolded protein response, reactive oxygen species, apoptotic cascade, and free radical scavenging on post-thaw viability using an in vitro hHPC model. HPC’s were selected as the model cell system as there is significant cell loss following cryopreservation. As such, any potential benefit of increased cell recovery would be a significant first step. It is important to note that this study focused on assessment of viable hHPC recovery, as this is a critical initial step in improving the overall outcome. This was investigated using samples cryopreserved in both extracellular (culture media) and intracellular type (Unisol™) cryopreservation solutions supplemented with varying concentrations of DMSO (5 and 10%) to assess the potential for broad-based application. Initial experiments focused on determining the impact of CIDOCD on viable hHPC recovery. Reports have shown that a 50 to 75% reduction in overall cell viability can result in the initial 24 h post-thaw period in numerous cell systems [22,94]. However, detailed reports on the extent of hHPC cell loss over this interval are limited [88,95,96,97,98,99,100]. Our data illustrated that there was a substantial level of hHPC loss over the initial 24 h recovery period (Figure 1). Specifically, it was found that an apparent >90% viable population 1 h post-thaw decreased to less than half that (between 20–40%) by 24 h post-thaw following hHPC cryopreservation using traditional culture media with 5 or 10% DMSO (Figure 1). This finding was consistent with previous reports that describe HPC and/or HSC post-thaw viability to range between 40 and 60% of pre-freeze controls with varying cryopreservation media formulations and cryoprotective agents, including 10% DMSO [64,88,95,96,97,98,99,100]. When samples were cryopreserved using Unisol™, overall hHPC viability at 24 h post-thaw increased to 60–65% depending on the DMSO concentration (Figure 2). This represented a significant increase in overall survival compared to the media + DMSO samples. Interestingly, Unisol + 5% DMSO resulted in a 20% increase in overall hHPC viability compared to media + 10% DMSO while using half the concentration of DMSO (Figure 2, U5 vs. M10). Our finding of improved HPC survival following cryopreservation in Unisol + DMSO is consistent with studies reporting improvements in overall post-thaw survival, CD34+ fraction, and population functionality following cryopreservation in other intracellular type cryopreservation solutions, including CryoStor CS5 and CS10 (5% and 10% DMSO, respectively) [51,101]. These studies also reported a similar or improved % recovery of the CD34+ cell fraction within the total surviving population. This correlates to an overall increased greater number of CD34+ cells recovered post-thaw from samples cryopreserved in an intracellular type solution, CryoStor, compared to extracellular type solutions. 

With the establishment of the significant level of hHPC loss over the initial 24 h post-thaw interval (media + DMSO = >60% decrease and Unisol™ + DMSO = ~40% decrease), we then investigated the impact of modulating various stress response pathways known to be activated following cryopreservation during the initial 24 h post-thaw recovery interval on overall hHPC survival. Initial studies focused on the addition of resveratrol into the recovery media, as several reports have indicated the role of free radical generation and damage following cryopreservation, as well as hypothermic storage of various cells and tissues [48,76,78,102,103,104,105,106,107,108,109]. Resveratrol supplementation had no significant impact on hHPC viability regardless of the cryopreservation solution (media or Unisol™) or DMSO concentration (5% or 10%) utilized (Figure 3 and Figure 4, ROS I conditions). Similar results (no benefit) were observed when the unfolded protein response inhibitor, salubrinal (UPR I), was incorporated into the recovery media. The one exception was in the case of M5 samples where salubrinal supplementation (UPR I) resulted in an overall ~10% increase in viability compared to the M5 parent samples. 

Studies were then expanded to examine the impact of caspase inhibition during recovery. Numerous studies have reported on the positive impact of caspase inhibitors during cryopreservation (e.g., added into the cryopreservation solution) [24,25,26,27,28,29,30,31,32,33,34] and, as such, we investigated the impact of targeted caspase inhibition during the recovery interval only. Supplementation of the recovery media with a pan-caspase inhibitor had little benefit on samples cryopreserved in media + DMSO (Figure 3). Interestingly, when this strategy was applied to samples cryopreserved in Unisol™ + DMSO, a significant increase in hHPC viability was observed (Figure 4). Post-thaw caspase modulation resulted in a ~15% increase in overall viability in U5 samples (U5 M + AI) and a ~11% increase in U10 samples (U10 M + AI). When comparing overall survival, 24 h post-thaw incubation in recovery media supplemented with the caspase inhibitor resulted in similar overall survival in both the U5 and U10 samples (75.3% and 73.8%, respectively, *p* = 0.64). Furthermore, this represented a near doubling of the overall survival compared to the samples cryopreserved in media + 10% DMSO (M10) with or without caspase inhibition (>73% vs. <42%, respectively, *p* < 0.001). 

Lastly, we investigated the impact of oxidative stress inhibition (ORS I) during recovery via recovery media supplementation with n-acetyl cystine. In contrast to the other agents, n-acetyl cystine supplementation to the recovery media resulted in a significant improvement in hHPC viability in samples cryopreserved in media + DMSO solutions. Post-thaw incubation of M5 samples in n-acetyl cystine resulted in an over doubling in hHPC overall viability (25% increase) compared to M5 samples alone (45% vs. 20%, respectively). Similar improvements were noted in M10 samples, where a ~20% increase in overall viability was found compared to M10 alone (63% vs. 43%, respectively). When the n-acetyl cystine post-thaw incubation strategy was applied to samples cryopreserved in Unisol™ + DMSO, a similar benefit was observed (Figure 4). For samples cryopreserved in Unisol™ + DMSO, post-thaw incubation in recovery media supplemented with n-acetyl cystine yielded a 16–18% increase in overall viability compared to non-supplemented Unisol™ samples (U5 and U10; media vs. M + ORS I conditions). Interestingly, in both U5 and U10 samples, post-thaw incubation in n-acetyl cystine resulted in an overall post-thaw viability (hHPC recovery) approaching 80% (78.0% and 78.8%, respectively). This represented a significant increase in post-thaw cell survival compared to any other condition evaluated. Equally important, studies focusing on recovery media supplementation with n-acetyl cystine revealed that this strategy resulted in a significant increase in hHPC viability at 24 h post-thaw, regardless of the cryopreservation solution (culture media or Unisol™) or DMSO concentration (5% or 10%) utilized. While promising, this study is not without limitations. First and foremost, this study focused on investigation of overall cell survival (viable cell number) following cryopreservation. While the results showed a significant increase in the recovery of viable cells following thawing, an analysis of functionality of the surviving hHPC population remains to be completed. Based on the positive findings reported herein, studies to quantitate the CD34+ cell fraction within the surviving population are now warranted. Another limitation is that this study was conducted on an in vitro cell model. To this end, initial observations of the CD34+ cells within the surviving HPC samples suggest a similar % fraction between samples cryopreserved in media or Unisol + DMSO and cultured in recovery media with or without n-acetyl cysteine supplementation. While preliminary, these initial findings are consistent with previous reports [51,88,95,96,97,98,99,100,101], and suggest that post-thaw conditioning may provide for increased recovery of total viable cells as well as maintenance of the CD34+ subpopulation, correlating to a higher number of viable CD34+ cells post-thaw. While encouraging, these observations are highly preliminary; therefore, further investigation must be completed prior to drawing any definitive conclusions. The in vitro nature provides a controlled environment for cell recovery. In vivo, following freezing and thawing, additional stressors associated with sheer stress (infusion), prolonged ischemia, engraftment, inflammatory response, etc., may provide additional destructive events, thereby increasing the level of cell death. As such, to further characterize the impact of post-thaw stress response modulation, homing and engraftment studies using an in vivo model need to be conducted. While further study is needed, the initial results of improved hHPC cell survival presented herein are an important first step in the development of improved strategies for recovering cryopreserved cells. Despite the limitations, the results are encouraging and support further exploration of post-thaw stress response modulation as a “back end” strategy to improve cell recovery in samples cryopreserved using various cryopreservation strategies (media, CPAs, protocols, etc.). To this end, additional studies by our group have confirmed the benefit of the post-thaw stress response modulation strategy in cell systems, including mesenchymal stem cells, endothelial, smooth muscle, and skeletal muscle cells [37,71,72,110,111]. 

## 5. Conclusions

Overall, the findings of improved cell survival presented in this study are an important initial step in understanding the potential impact of modulating stress pathway activation associated with cryopreservation during the recovery interval. This study also demonstrated that in order to gain an accurate picture of viability, the utilization of more discriminating assays, such as metabolic activity assessment, and extended recovery intervals (24 h vs. 1 h) is necessary. Furthermore, the results confirmed the reported benefits of intracellular-like solutions for improving overall hHPC survival following cryopreservation [30,53,57,60,101]. Most importantly, the results demonstrated that significant improvements in post-thaw hHPC recovery can be attained regardless of the cryopreservation media utilized through the use of specialized recovery media supplemented with various cell stress modulating agents. 

While promising, a more complete analysis of the cell stress pathway signaling cascades activated and the impact of their modulation post-thaw will be critical for further improving hHPC recovery. In this regard, hHPC cryopreservation includes a number of different cell stresses such as thermal shifts, hypoxia, and mechanical stress, among others, each of which may activate a different set of cell stress pathways. Furthermore, as described above, based on the positive findings reported herein, characterization of differentiation capability, CD34+ fraction, ability to home, etc., of hHPC’s post-treatment must be completed to fully understand the impact of our findings. As the therapeutic potential of hHPC’s grows, the ability to successfully cryopreserve and deliver these cells will continue to play an increasing role in therapeutic success. The ability to modulate stress responses post-thaw could allow for optimal retention of viability and functionality. While work remains, these results represent an important step forward in the development of improved cryopreservation processes for use in discovery science, and commercial and clinical settings. 

## Data Availability

Not applicable.

## Abbreviations

CIDOCD, Cryopreservation Induced Delayed Onset Cell Death; CPA, Cryoprotective Agent; DMSO, Dimethyl Sulfoxide; hHPC, Human Hematopoietic Progenitor Cells; M5, Media + 5% DMSO; M10, Media + 10% DMSO; U5, Unisol + 5% DMSO; U10, Unisol + 10% DMSO; A, apoptotic caspase cascade; UPR, unfolded protein response; OSR, mitochondrial oxidative stress response; ROS, reactive oxygen species; UPR I, salubrinal; AI, Apoptotic Inhibitor; ROS I, Resveratrol; ORS I, n-acetyl cystine; GMP, Good Manufacturing Practice.
